# Development and validation of a semi-automated and unsupervised method for femur segmentation from CT

**DOI:** 10.1038/s41598-024-57618-6

**Published:** 2024-03-28

**Authors:** Alessandra Aldieri, Riccardo Biondi, Antonino A. La Mattina, Julia A. Szyszko, Stefano Polizzi, Daniele Dall’Olio, Nico Curti, Gastone Castellani, Marco Viceconti

**Affiliations:** 1 PolitoBIOMedLab, Department of Mechanical and Aerospace Engineering, Politecnico di Torino, Turin, Italy; 2 Medical Technology Lab, IRCCS Istituto Ortopedico Rizzoli, Bologna, Italy; 3 IRCCS Bologna - Istituto delle Scienze Neurologiche di Bologna, Bologna, Italy; 4 Department of Medical and Surgical Sciences, Alma Mater Studiorum - University of Bologna, Bologna, Italy; 5 Department of Industrial Engineering, Alma Mater Studiorum - University of Bologna, Bologna, Italy; 6 Department of Physics and Astronomy, Alma Mater Studiorum - University of Bologna, Bologna, Italy

**Keywords:** Bone and joint enhancement filter, CT, Femur segmentation, Finite element model, Graph-cut, Semi-automated segmentation, Bone imaging, Engineering, Biomedical engineering

## Abstract

Quantitative computed tomography (QCT)-based in silico models have demonstrated improved accuracy in predicting hip fractures with respect to the current gold standard, the areal bone mineral density. These models require that the femur bone is segmented as a first step. This task can be challenging, and in fact, it is often almost fully manual, which is time-consuming, operator-dependent, and hard to reproduce. This work proposes a semi-automated procedure for femur bone segmentation from CT images. The proposed procedure is based on the bone and joint enhancement filter and graph-cut algorithms. The semi-automated procedure performances were assessed on 10 subjects through comparison with the standard manual segmentation. Metrics based on the femur geometries and the risk of fracture assessed in silico resulting from the two segmentation procedures were considered. The average Hausdorff distance (0.03 ± 0.01 mm) and the difference union ratio (0.06 ± 0.02) metrics computed between the manual and semi-automated segmentations were significantly higher than those computed within the manual segmentations (0.01 ± 0.01 mm and 0.03 ± 0.02). Besides, a blind qualitative evaluation revealed that the semi-automated procedure was significantly superior (*p* < 0.001) to the manual one in terms of fidelity to the CT. As for the hip fracture risk assessed in silico starting from both segmentations, no significant difference emerged between the two (*R*^2^ = 0.99). The proposed semi-automated segmentation procedure overcomes the manual one, shortening the segmentation time and providing a better segmentation. The method could be employed within CT-based in silico methodologies and to segment large volumes of images to train and test fully automated and supervised segmentation methods.

## Introduction

Nowadays, a large portion of the morbidity, mortality and health expenditure in a progressively ageing population is related to the musculoskeletal system^1^. Bone fracture is one of the most common age-related occurrences, caused by the interplay of several factors such as an increased propensity to fall, the decreased mechanical competence of the bone tissue and the possible presence of pathologies like osteoporosis and arthrosis. The main skeletal districts commonly interested by a fracture are the hip, the spine and the wrist^2^. Hip fractures are particularly severe, as they place a considerable burden on the quality of life and increase mortality, which spans from 10 to 40% in the first year after the fracture event^3^. The current gold standard adopted to predict the risk of a hip fracture is the areal Bone Mineral Density (aBMD) computed through the Dual-energy X-ray absorptiometry (DXA) technique, which might also be used in combination with other epidemiological risk factors such as sex, age, weight, alcohol intake, etc.^4^. Nevertheless, despite being correlated with hip fracture incidence, aBMD is a fully phenomenological surrogate of the hip fracture risk, not able to include the three-dimensional geometry of the femur as well as the internal architecture and material properties distribution of the bone. In fact, only one-third of the lower trauma fractures can be explained by aBMD^5^. Quantitative Computed Tomography (QCT)-based Finite Element (FE) models, instead, have proved to enhance the fracture risk prediction. In^6–9^, the use of patient-specific FE models was shown to outperform aBMD accuracy in predicting the actual hip fracture risk. In fact, patient-specific FE models not only allow to account for the specific geometry and local material properties of the femur, but also to investigate the realistic wide range of possible boundary conditions acting on the femur during a fall.

The first step to build a FE model based on clinical QCT images is represented by the QCT images segmentation, through which the subject’s femur geometry is extracted. The segmentation task is not straightforward, especially due to the presence of a thin hip joint space separating the femur bone from the pelvis. This condition implies a significant partial volume effect, which makes the proximal femur segmentation challenging even in physiological conditions. Moreover, pathologies such as osteoarthritis and osteoporosis make it even harder to separate the head of the femur from the iliac bones. For these reasons, the segmentation procedure is performed almost fully manually in most cases. However, the manual segmentation procedure is time-consuming, subject to the operator’s expertise and difficult to reproduce. In addition, it hinders the desired clinical application of FE-based fracture predictors, which would be fostered by the full automatization of their development starting from clinical images. Therefore, the adoption of automated segmentation methods not requiring any user interaction could foster the uptake of these in silico tools within the clinical practice.

As a matter of fact, most of the recently proposed fully automated segmentation methodologies are supervised^10–15^, meaning that they are based on pre-existing labelled datasets. Such supervised methodologies can be based on previously built statistical shape models which are employed to segment new femur instances^13,16^, but more commonly automatic pipelines based on deep learning techniques are proposed^11,17^, with Convolutional Neural Network (CNN)-based techniques widely presented recently^12,18–22^. Nevertheless, machine-learning based techniques require the availability of large amounts of labelled data for training^23^, which is not straightforward: the collection of segmented CT scans is usually performed manually, and it can be time-consuming, operator- dependent, or even impossible^24^. Thus, halfway are semi-automated methodologies, which require minimal user interaction and can therefore be faster and more reproducible than fully manual segmentation methods though being completely unsupervised, i.e., not requiring a labelled dataset. These methods could also represent promising options for creating large, labelled datasets, which might eventually be used to train and test supervised and fully automated methods.

In this context, this work aimed to implement and validate a semi-automated and unsupervised pipeline to segment femur CT scans. Such a pipeline would not require training while requiring the intervention of a user to complete the images segmentation. Inspired by the work of Besler^25^ and Krcah^26^, a pipeline based on a bone enhancement filter and graph cut was implemented. The outcomes of the semi-automated methodology were here compared with the outcomes of the manual segmentations on the same subjects, not only in terms of femur geometry differences, segmentation reproducibility, and blind visual assessment, but also in terms of the outcomes of a biophysical digital twin called Bologna Biomechanical Computed Tomography at the hip (BBCT-hip), which was recently proposed as an in silico methodology to predict the risk of a hip fracture upon falling^27,28^. BBCT-hip estimates the Absolute Risk of Fracture at time x (ARFx) by orchestrating a stochastic analytical model, which predicts one million possible impact forces due to a fall on the side, and a QCT-based FE model of the femur, which calculates the force necessary to fracture it (load to failure). The comparison between the two segmentations approaches based on the outcomes of an in silico methodology beyond geometry-based metrics was considered pivotal, as the segmentation is not performed for its own sake, but rather represents the essential starting point of the fracture risk prediction in silico.

## Methods

### Semi-automated segmentation procedure

**Figure 1:**
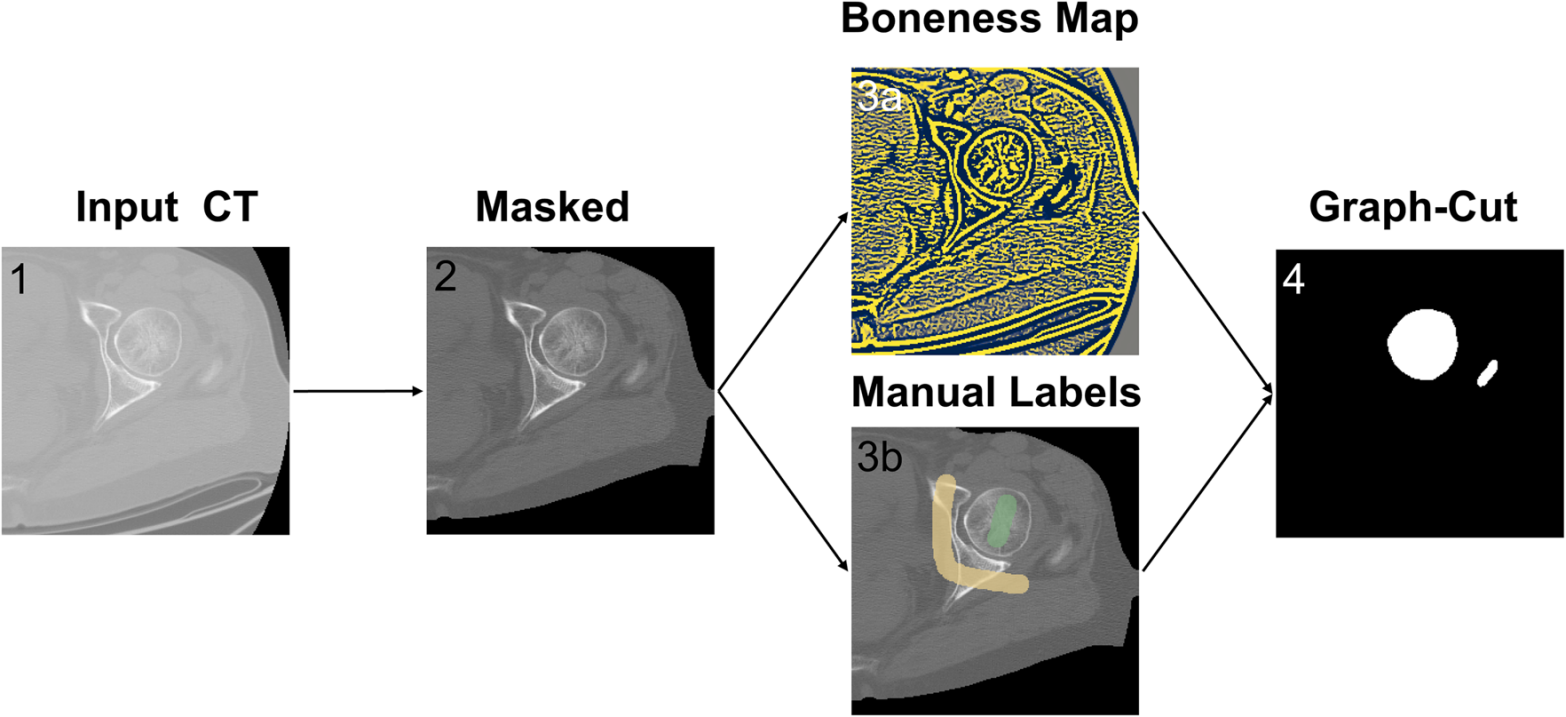
A schematic overview of the semi-automated segmentation pipeline being proposed. It displays the successive steps of the pipeline from left to right: (1) one slice of the original CT scan; (2) body segmentation step: the body region is isolated disregarding the rest; (3a) the BJE filter is applied to the CT scan to enhance the cortical bones and suppress the joint region and other tissues; (3b) in parallel, the user manually labels a few CT slices as either femur bone (shown in green) or background (shown in yellow); (4) the graph cut is implemented to achieve binary segmentation.

**Figure 2:**
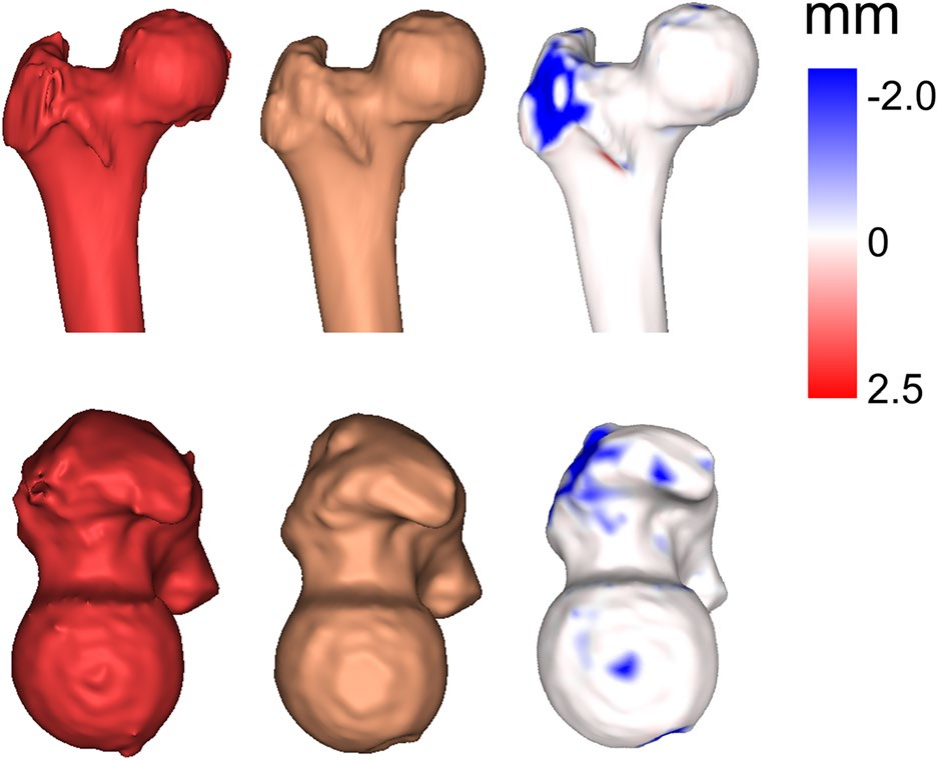
The three-dimensional geometry of one proximal femur, resulting from the semi-automated procedure. The geometry obtained directly from the graph-cut is shown in red, while in brown the same geometry is displayed after the manual refinement step. Eventually, on the right a distance map is displayed which illustrates the impact of the manual refinement.

The semi-automated and unsupervised segmentation framework, implemented in Python and available in Open Access on GitHub (https://github.com/RiccardoBiondi/FemurSegmentation), was based on the cortical bone and joint enhancement (BJE) filters and on Boykov and Jolly’s graph-cut^29^ inspired by the works of Krcah^26^ and Besler^25^. The semi-automated segmentation pipeline consists of three phases, which will be better detailed in the following and presented in Fig. 1: (1) preliminary body region segmentation, (2) BJE filter and per-voxel term initialisation, (3) final graph-cut-based segmentation. Eventually, a manual refinement step can be included (Fig. 2).

First, within the body segmentation phase, a threshold above -400 HU was applied to the CT scans to define a region of interest (ROI), excluding all the air and padding values of the CT. This decreased the computational time of the graph-cut and BJE filter, as it reduced the number of voxels to consider in the next steps. Subsequently, the BJE filter was computed, and, in parallel, a few CT slices were manually labelled by an operator to define foreground (i.e., the femur bone) and background regions. Afterwards, the graph-cut-based segmentation was performed, similarly to what was done in^30^. Eventually, the manual refinement step allowed the removal of possible localised errors (Fig. 2). Overall, the operator intervention took place in two stages, labelling and refinement, which involve short tasks that do not require excessive training. On average, executing the whole semi-automated procedure required 10–20 min to run, considering that the manual intervention parts are the most time consuming. Nevertheless, if only the graph-cut implementation is considered without considering any manual intervention, its average execution time was 54 ± 4 s, obtained by repeating the segmentation 13 times for 10 femurs. In the following the BJE filter adoption and graph-cut algorithm are described in detail.

#### Bone and joint enhancement (BJE) filter

**Figure 3:**
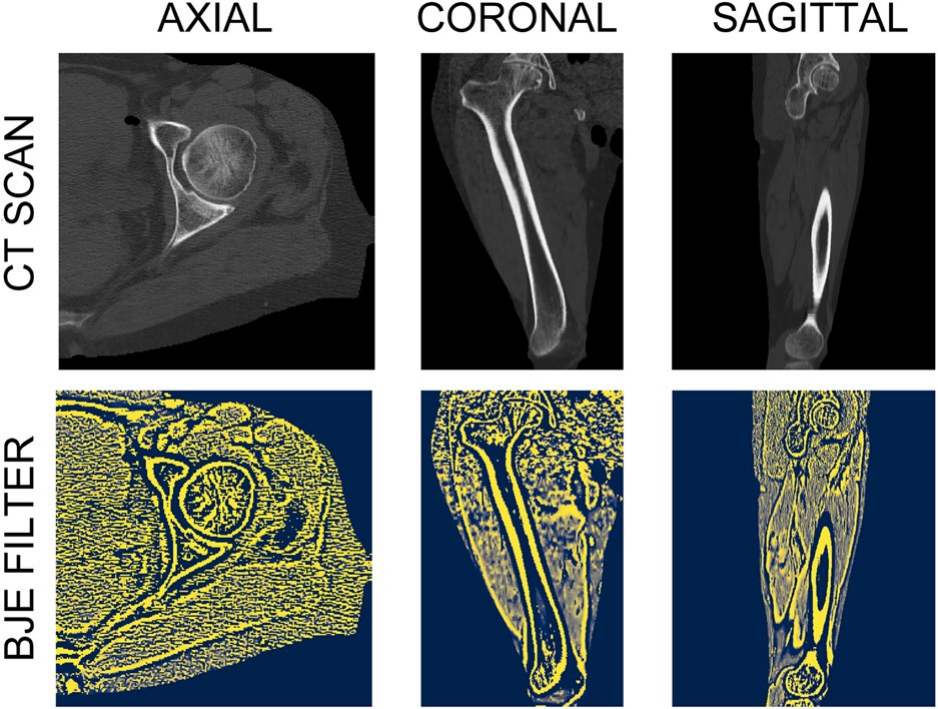
BJE filter: CT scans before (upper panel) and after (lower panel) its application. The upper panel shows the original CT image in HU, while the lower panel displays the Boneness map, which is obtained by applying the BJE filter. This filter generates a range of values from − 1 to 1, with − 1 representing the joints and 1 representing the cortical bones. The Boneness map highlights the joint and cortical areas more prominently than what is seen in the original scan.

The BJE filter is based on the eigenvalues of the local Hessian matrix computed on the voxels Hounsfield Units. The local Hessian matrix is computed by convolving the original 3D CT with the second and cross derivatives of a Gaussian. In this way, a 3 × 3 Hessian matrix is obtained, containing the second order and cross derivatives in each direction (Axial, Sagittal and Coronal) for each voxel in the CT volume. The eigenvalues analysis allows the extraction of the principal directions in which the local second-order structure of the image can be decomposed. More in detail, it maps a spherical neighbourhood centred around the voxel of interest onto an ellipsoid in the hessian image, where the eigenvalues represent the ellipsoid radius. Let $|{\lambda }_{3}| > |{\lambda }_{2}| > |{\lambda }_{1}|$ be the eigenvalues of the local Hessian Matrix for the voxel *p*. BJE filter application outcome, referred to as Boneness map (*p*), was computed as follows:

1 $$ BJE\left( p \right) = - sign\left( {\lambda_{3} } \right)e^{{ - \frac{{R_{bones}^{2} }}{2}}} \left( {1 - e^{{ - 4R_{noise}^{2} }} } \right), $$

where ${R}_{bones}=\frac{|{\lambda }_{1}{\lambda }_{2}|}{{\lambda }_{3}^{2}}$ and ${R}_{noise}=\frac{\left|{\lambda }_{1}\right|+\left|{\lambda }_{2}\right|+|{\lambda }_{3}|}{T}$. $T$, which acts as a regularization factor, is computed as the mean of the absolute value of the eigenvalues across the whole image. According to Krach^26^, the sign of the largest eigenvalue allows to enhance both the cortical structure and the joint space (Fig. 3): in this way, it is possible to increase the contrast between the bone and the hip joint. The *R*_*bones*_ term discriminates between tube-sheet-like structure, i.e., the cortical bones, and blob-like structure, i.e., the surrounding tissues. The *R*_*noise*_ term allows local noise suppression. In Fig. 3, different views of the CT with the resulting Boneness map are presented. The cortical bone (bright region) is enhanced, while the joints and the tissues surrounding the bones get suppressed. Table 1 displays the pseudocode related to the implementation of the BJE filter.

**Table 1:**
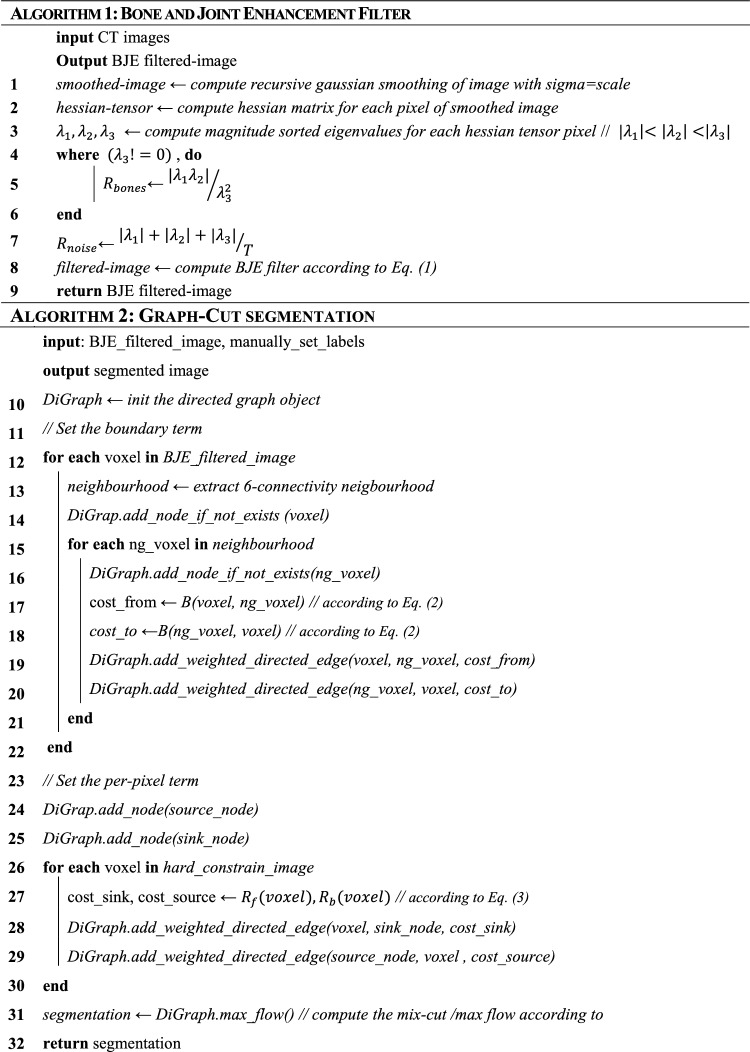
Pseudocode for the Bone and Joint enhancement (BJE) filter (Algorithm 1) and for the Graph-cut implementation (Algorithm 2).

In the pseudocode the main step followed during the python implementation of the BJE filter and Graph-cut are highlighted. After getting the CT scans as inputs, the boundary term (lines 3–13) and the per pixel term (lines 17–21) are set and the BJE filtered image is obtained. The BJE filtered image is then taken as input by the Graph-cut, which sets the boundary and per-pixel terms and eventually runs the min-cut/max flow (line 31), returning the binary segmentation as outcome.

#### Graph-cut framework

Graph-Cut is an energy minimization technique based on the combinatorial graph theory^29^. The method aims to minimize a cost function composed of two terms: a boundary term (*B*) and a per-voxel term (*R*). The first term (*B*) is the penalty of classifying two neighbouring voxels, referred to as *p* and *q*, in different classes; the second term (*R*) specifies the cost of assigning a voxel to a specific class (either background or foreground). The boundary term was computed as specified in Eq. (2), based on the Boneness map obtained by the application of the BJE filter on the CT image.

2 $$ B\left( {p,q} \right) = \left\{ {\begin{array}{*{20}l} {e^{{ - \lambda \frac{{\left( {BJE\left( p \right) - BJE\left( q \right)} \right)^{2} }}{{2\sigma^{2} }} }} } \hfill & {if\, BJE\,\left( p \right) > BJE\left( q \right)} \hfill \\ \lambda \hfill &{otherwise} \hfill \\ \end{array} } \right. $$

with σ and $\lambda$ being a noise and a weighting term, respectively^29^. The boundary term in Eq. (2) penalizes the discontinuities between voxels with values significantly similar, i.e., $(BJE(p)-BJE(q)) < \sigma$. On the contrary, if the difference between the two voxels is high, e.g., $(BJE(p)-BJE(q)) >> \sigma$, the penalty to assign them to different classes is minimal. Therefore, setting the value of σ too high would cause all the voxels to be considered very similar, thus making it nearly impossible to assign them to different classes^29^.

**Figure 4:**
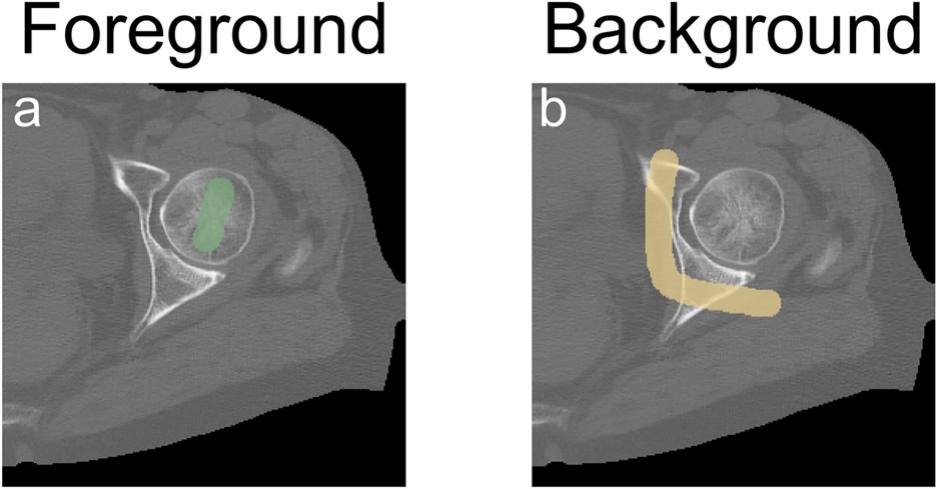
Example of the labelling performed by the operator. Left: in green, labels for the foreground voxels. Right: in yellow, labels for the background. Limited and not necessarily precise labelling was sufficient to initialise the graph-cut.

The per-voxel term *R*, instead, consists of two parts: one (${R}_{b}$) specifies the cost of classifying a voxel *p* as background, and the other (${R}_{f}$) the cost to classify the voxel as foreground. They were defined as specified in the following:

3 $$ R_{b} \left( p \right) = \left\{ {\begin{array}{*{20}c} {\lambda \,if\, p \in bkg} \\ {0 \,if\, p \in frg} \\ {1\, otherwise} \\ \end{array} } \right. $$

4 $$ R_{f} \left( p \right) = \left\{ {\begin{array}{*{20}c} {\lambda \,if\, p \in frg} \\ {1 \,if\, p \in bkg} \\ {0\, otherwise} \\ \end{array} } \right. $$

where $frg$ and $bkg$ refer to the foreground and the background labels, respectively. To determine whether a voxel *p* belongs to the foreground or the background, a trained and expert operator sparsely labelled a few CT slices, as reported in Fig. 4. These labels were used in Eqs. (3) and (4) to determine if a voxel belonged to the foreground (i.e., is labelled as foreground, with ${R}_{b}\left(p\right)=0$ and ${R}_{f}\left(p\right)=\lambda$), to the background (i.e., is labelled as background, with ${R}_{b}\left(p\right)=\lambda$ and ${R}_{f}\left(p\right)=1$) or if no information was provided (${R}_{b}\left(p\right)=1$ and ${R}_{f}\left(p\right)=0$).

The cost function $E$ minimised by graph-cut was defined as:

5 $$ E\left( A \right) = \mathop \sum \limits_{p \in A} R\left( p \right) + \lambda \mathop \sum \limits_{p,q \in N} B\left( {p,q} \right) $$

where $\sum_{p\in A}R\left(p\right)=\sum_{p\in A}{R}_{f}\left(p\right)+\sum_{p\in A}{R}_{b}\left(p\right)$. $A$ refers to the analysed image, $N$ is the set of all neighbouring voxels *q* for each voxel *p* belonging to the image *A* and λ acts as a weighting factor, increasing (or decreasing) the weight of the boundary term on the segmentation. The min-cut/max-flow algorithm^29^ was adopted to identify the segmentation that minimises the cost function. The parameters σ and λ were set to 0.25 and 100 respectively. A detailed discussion about how those parameters affected the graph-cut segmentation is provided in Section 1 of the Electronic Supplementary Information file. In addition, Table 1 also provides the pseudocode related to the graph-cut implementation.

### Manual segmentation procedure

**Figure 5:**
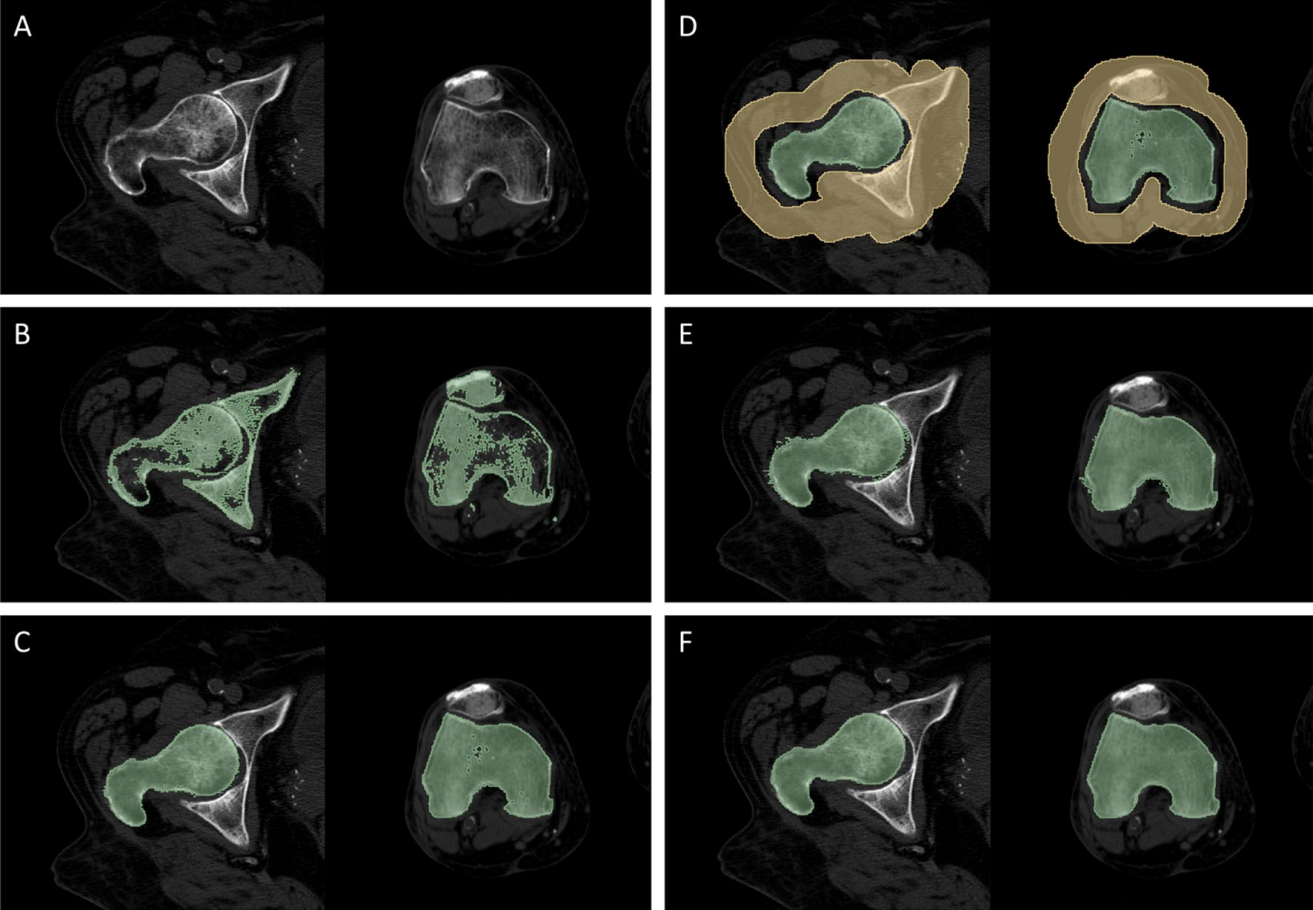
Manual segmentation process. (**A**) starting CT image; (**B**) a HU-based threshold-is applied to the CT slices, resulting in the depicted mask (green); (**C**) the femur bone mask after manual intervention aimed to remove the other bones (e.g., pelvis, patella) from the mask; (**D**) the creation of a surrounding tissue mask (yellow) comprising what it is not the femur bone of interest, needed to initialise the GrowCut algorithm; (**E**) the result of the GrowCut-based region growing procedure; (**F**) the final segmentation mask after manual refinement and smoothing.

The manual segmentation procedure was implemented in 3D Slicer (version 5.0.3) and it is presented in its main steps in Fig. 5. It started from a thresholding step, where a mask was created by setting a threshold dependent on each patient’s CT scan parameters to isolate only the HU values corresponding to the femur bone tissue (Fig. 5B). Further refinement through manual intervention, especially in complex areas like the hip joint space region, was crucial due to the variations in bone density and overlapping structures. This involved slice-by-slice evaluation by the operator to distinguish the femur head from the iliac bone and the condyles from the patella (Fig. 5C). Subsequently, another mask was created by selecting the other tissues surrounding the femur, such as muscles, tibia or pelvis, with special attention paid to the hip joint space (Fig. 5D). Following this, region growing was carried out thanks to the GrowCut algorithm implemented in 3D Slicer^31^, which allowed to finalise the femur bone segmentation (Fig. 5E). Eventually, a last manual refinement step was performed, followed by smoothing using a median filter (kernel size of 2 mm) (Fig. 5F). On average, the implementation of the whole manual procedure required 60 min to be run.

### Evaluation of semi-automated segmentation

Aiming to compare the presented semi-automated pipeline with the manual segmentation procedure, a total of ten subjects were selected from the HipOp registry in Rizzoli Orthopaedic Institute and their CT images were segmented according to both approaches. All methods were carried out in accordance with relevant guidelines and regulations, informed consent was obtained from all subjects and approval was granted from the Rizzoli Orthopaedic Institute Ethics Committee (CE AVEC 731/2020/Oss/IOR). The subjects involved were all post-menopausal women, older than 66 years at the time of the CT scan, with height and weight ranging from 134 to 165 cm (160.3 ± 7 cm) and from 41 to 71 kg (65.5 ± 11.8 kg) respectively. Two of them did experience a hip fracture after the CT scan. Six of the analysed femurs were left, while the remaining four were right. The images were acquired in axial direction using the following parameters: tube voltage at 120 kVp, tube current at 150–200 mA and a focal spot of 0.7 mm. The slice thickness of the acquired images ranged from 2.5 to 3.0 mm and the voxel spacing (isotropic in the other two directions) was between 0.66 and 0.78 mm. Both the manual and semiautomated segmentation procedures were performed by an experienced operator. Aiming to compare the two methodologies in terms of reproducibility, the operator performed both segmentations four different times. Two aspects were considered for the comparison: the resulting segmentation geometry and its impact on the ARF0 prediction. In both cases, intra-segmentation differences, i.e., differences related to segmentations obtained with the same method, and inter-segmentation differences, i.e., differences related to segmentations obtained with the different techniques, were assessed.

#### Geometry

Aiming to compare the segmentations in terms of their resulting geometry, a quantitative comparison based on distance metrics and a blind visual comparison were employed. For the quantitative comparison, three different similarity measures were used. The first was the distance between the segmentations computed using the Difference Union Ratio (*DUR*), also known as Jaccard Distance. This similarity measurement considers the difference (in terms of union and intersection ratio) between two binary volumes. Given two segmentation volumes, *A* and *B*, the *DUR* is defined as:

6 $$ DUR\left( {A,B} \right) = 1 - \frac{A \cap B}{{A \cup B}} = \frac{A \cup B - A \cap B}{{A \cup B}} $$

The distance between each couple of binary segmentations was also computed in terms of the Hausdorff Distance (*HD*) and the Average Hausdorff Distance (*AHD*). Given two segmentations *A* and *B* the *HD* and *AHD* were calculated as:

7 $$ HD\left( {A,B} \right) = max\left( {e\left( {A,B} \right),e\left( {B,a} \right)} \right) $$

8 $$ AHD\left( {A,B} \right) = mean\left( {e\left( {A,B} \right),e\left( {B,a} \right)} \right) $$

where $e\left(A,B\right)=\underset{a\in A}{\mathit{min}}\left(d\left(a,B\right)\right)$ and $d\left(a,B\right)=\underset{b\in B}{\mathit{inf}}(d\left(a,b\right))$, with $d\left(a,b\right)$ being the Euclidian distance between points *a* ∈ *A* and *b* ∈ *B*. Thus, the *HD* measures how far two subsets of a metric space, i.e., the two segmentations, are from each other at most. While due to its definition, the *HD* is sensitive to worst-case errors, localised in small areas, its averaged variant (*AHD*) is not, turning out to be more robust against isolated error peaks.

The blind visual comparison, instead, involved four expert trained operators who compared the segmentations superimposed to the corresponding CT image slice by slice and established, if possible, the best segmentation based on its fidelity to the CT bone contour. This visual comparison was carried out blindly, aiming to avoid biases, and was conducted using a custom-made software (https://github.com/RiccardoBiondi/segmentation_blind_evaluation). Since each femur had been segmented four different times with both the manual and semi-automated method, a representative segmentation was obtained from the four available for each methodology using the STAPLE algorithm^32^. Besides, the blind visual comparison was limited to the proximal region, since that was the region of interest considered within BBCT-hip methodology and, in addition, also the most critical region to be segmented.

#### ARF0 computation

The semi-automated and manual segmentation procedures were also compared in terms of the fracture risk predicted by the BBCT-hip digital twin solution. BBCT-hip pipeline was implemented starting from all the segmented femur geometries, and the absolute risk of fracture (ARF0) was computed for each of them. More details concerning the BBCT-hip pipeline can be found in the Electronic Supplementary Information file.

#### Statistical analysis

Aiming to compare the semi-automated and the manual segmentations, the distance metrics mentioned before (*DUR*, *HD*, *AHD*) were computed within the repeated manual and semi-automatic segmentations (intra-segmentation) as a variability measure of and across the semi-automated and manual segmentations (inter-segmentation), considering all possible pairings. The Mann–Whitney U-test was conducted to identify significant differences between the intra- segmentation and inter-segmentation metrics. The same test was also employed to compare the ARF0 values obtained starting from the segmentations output by the two methodologies.

In addition, also a linear regression analysis between the intra-manual (independent variable) and inter-segmentation (dependent variable) distance metrics and a linear regression analysis between the manual (independent variable) and semi-automated (dependent variable) segmentation-based ARF0 values were carried out. The null hypothesis for the linear regression model was the absence of significant linear correlation between the dependent and independent variables: if the null hypothesis could not be rejected (*p* > 0.05), there would be no statistical evidence that the two segmentation techniques provided the same result.

Furthermore, a patient-wise analysis was also performed to establish whether the segmentation methodologies comparison could have been affected by some subjects’ peculiarities, such as femur anatomy or possible pathological conditions. We considered each patient as a single group of measures, where each subject was represented by the mean of the three geometrical metrics (*DUR*, *HD*, *AHD*) computed between each manual segmentation and the remaining ones (for the intra-method variability) and between each manual segmentation and all the semi-automated ones (for the inter-method variability). Therefore, four measurements were obtained as a measure of the intra-manual segmentation variability and four corresponding measurements were obtained as estimates of the inter-segmentation variability for each patient. Hence, generalised linear mixed models (GLMM) were used to assess the differences between groups, modelled as random effects. GLMM were built considering each patient as a different group of measurements, the manual intra- segmentation values as the dependent variables and the inter- segmentation values as the independent ones. Four different models were implemented:

1. $Y_{i,j} = \beta_{0} + \beta_{1} X_{i,j} + \gamma_{0,j} + \gamma_{1,j} X_{i,j} + \epsilon_{i,j}$ *with correlated random effects*
2. $Y_{i,j} = \beta_{0} + \beta_{1} X_{i,j} + \gamma_{0,j} + \gamma_{1,j} X_{i,j} + \epsilon_{i,j}$ *with non-correlated random effects* $(\gamma_{0,j} \bot \gamma_{1,j} )$
3. $$Y_{i,j} = \beta_{0} + \beta_{1} X_{i,j} + \gamma_{0,j} + \gamma_{1,j} X_{i,j} + \epsilon_{i,j}$$
4. $$Y_{i,j} = \beta_{0} + \beta_{1} X_{i,j} + \gamma_{0,j} + \gamma_{1,j} X_{i,j} + \epsilon_{i,j}$$

Where $j = {j}^{th}$ group, $i = {i}^{th}$ individual, ${Y}_{j}$ and ${X}_{j}$ dependent and independent variables respectively, ${\beta }_{0}$ the fixed effect intercept, ${\beta }_{1}$ the fixed effect slope, ${\gamma }_{0j}$ the random effect intercept, ${\gamma }_{1j}$ the random effect slope and ${\epsilon }_{i,j}$ the residuals. The likelihood ratio test was adopted to compare each model with the others, under the null hypothesis of comparable goodness of fit.

#### Blind evaluation analysis

The blind evaluation analysis consisted in labelling the CT slices as Manual, Semi-automated or None, according to that which segmentation was judged the best by the operator, for all the operators involved in the analysis. The assessments were merged using a majority voting procedure, i.e., considering the frequency of each label for each patient. The frequency distributions of the labels were obtained for each patient and compared using the one-sided Wilcoxon test.

## Results

### Geometry

**Figure 6:**
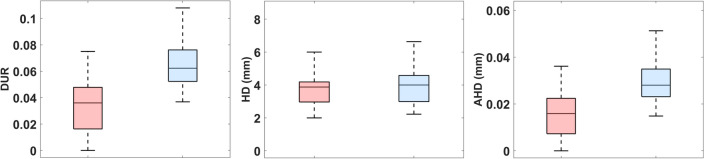
Boxplots comparing the distributions of the intra-manual (in pink) and inter-segmentation (in light blue) metrics computed: the DUR, the HD and the AHD.

The Mann–Whitney U-test revealed statistically significant differences between the intra-manual and inter- segmentation metrics as far as the *DUR* and *AHD* (*p* < 0.001) are concerned, with the inter-segmentation values being higher than the intra-segmentation ones. The *HD* did not show any significant differences between the two. In Fig. 6 the boxplots of the three distances metrics are shown. Figure 2s in the Electronic Supplementary Information file depicts the comparison between the intra-manual and intra-semi- automated distance metrics distributions.

In Table 2, slope, intercept, *p*-value and *R*^2^ for the *DUR*, the *HD* and the *AHD* metrics are reported. As visible, the null hypothesis could be rejected only for the HD metric. For the sake of brevity, the corresponding plots are shown in the Electronic Supplementary Information file (Fig. 3s).

**Table 2:** Linear regression results for each tested metric.

Metric	Intercept (µ ± σ)	Slope (µ ± σ)	*p*-value	${R}^{2}$
DUR	0.06 ± 0.01	0.02 ± 0.36	0.97	$2\times {10}^{-4}$
HD	0.37 ± 1.0	0.96 ± 0.27	0.01	0.61
AHD	0.027 ± 0.007	0.20 ± 0.4	0.64	0.03

The regression lines intercept and slope are reported with a standard error of one standard deviation. The obtained *p*-value and ${R}^{2}$ are also included.

In Table 3 the results of the tested generalised linear mixed models for the three distance measures are shown, with the corresponding likelihood ratio test *p* -values. The addition of the random intercept (3 vs 4) turned out to significantly affect (*p*-value < 0.05) the goodness of fit of the models. In contrast, the random slope contribution was significant only in the case of the *HD*. In Table 1s and 2s in Electronic Supplementary Information file the values of the random effects of the selected model for each metric are also reported.

**Table 3:** *p*-values for each metric and each compared GLMM obtained from the likelihood ratio test.

Compared Models	*p*-value DUR	*p*-value HD	*p*-value AHD
1 versus 2	0.21	0.013	0.074
2 versus 3	0.60	0.02 (1 vs 3)	1.00
3 versus 4	$3.00\times {10}^{-9}$	Not tested	$6.00\times {10}^{-9}$

For the Hausdorff Distance (HD) case model 2 versus 3 and 3 versus 4 were not tested since model 1 turned out to have a significantly higher likelihood with respect to model 2, highlighting the significant contribution of the correlated random effects.

**Figure 7:**
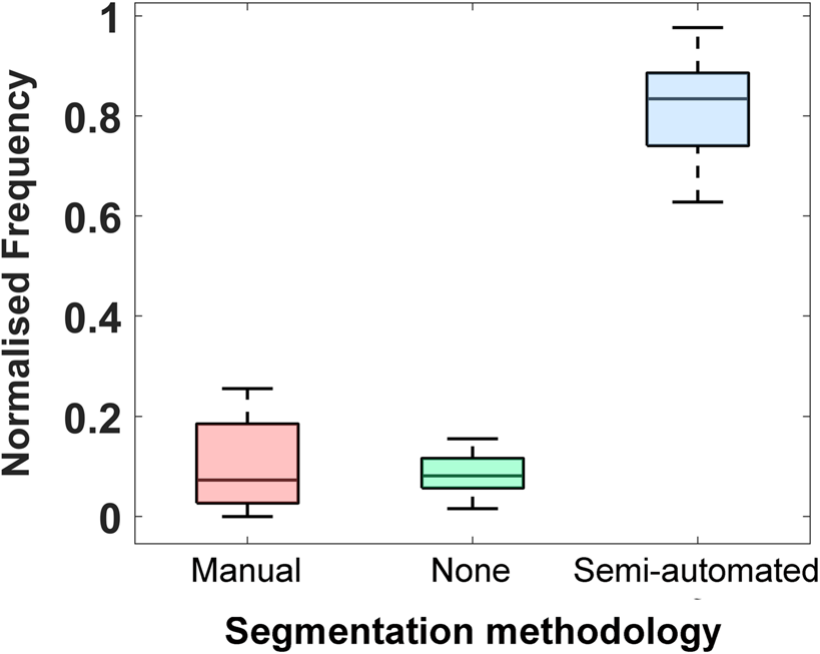
Boxplot reporting the outcomes of the blind visual comparison between the two segmentation methodologies. The frequency at each class (Manual, None, Semi-Automated) has been chosen is reported.

Figure 4s in the Electronic Supplementary Information file depicts the GLMMs regression on the considered distance metrics. In Fig. 7, the distributions resulting from the blind visual comparison are reported, according to the frequency of the labels selected by the experts. As visible, the median of the distribution corresponding to the Semi-automated label turned out to be significantly higher than the other two (*p*-value < 0.001), with no significant difference between the Manual and the None distributions.

### BBCT-hip ARF0

**Figure 8:**
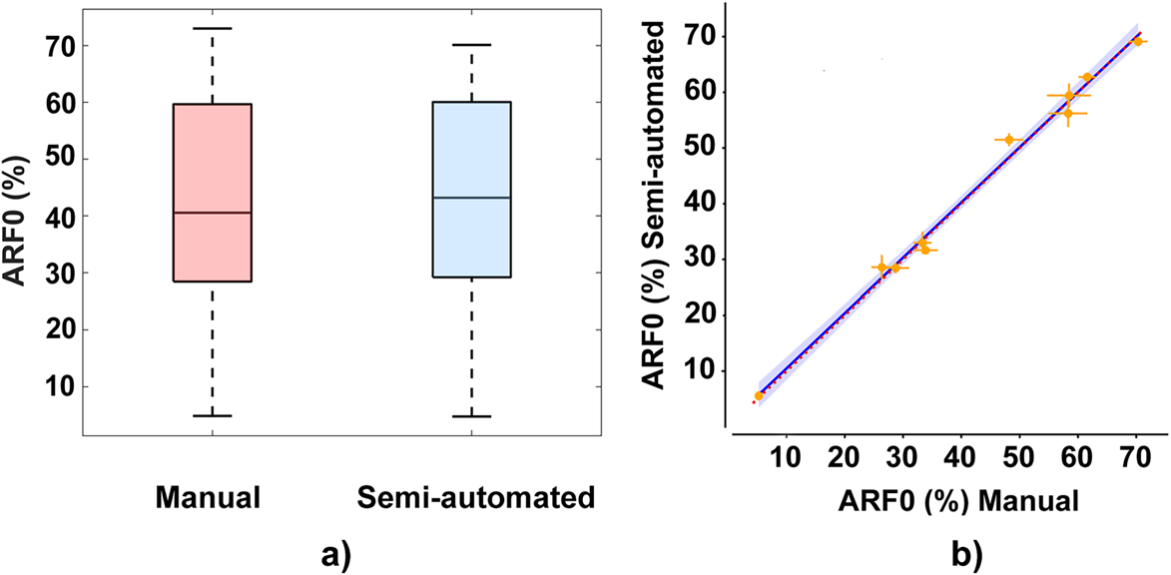
(**a**) Boxplots comparing the distributions of the ARF0 values obtained adopting the manual segmentation procedure and those obtained adopting the semi-automated segmentation methodology. (**b**) Linear regression between the ARF0 prediction for each patient resulting from the two segmentation procedures. The error bars report the standard deviation of the estimated values. In blue the estimated regression line with the 95% confidence interval is reported, in red the bisector line as reference.

As far as BBCT-hip’s main outcome is concerned, namely ARF0, Fig. 8 highlights the strong correlation between the ARF0 values estimated starting from the outcomes of the two segmentation methodologies. No significant differences could be found between the two groups (Fig. 8a) and the linear regression carried out yielded a *R*^2^ value of 0.99, highlighting the strong linear correlation between the ARF0 values predicted starting from the manual and semi- automated methodologies (Fig. 8b).

## Discussion

Implementing fully automated in silico pipelines able to support the clinical decision would strongly foster the adoption of in silico medicine in the clinical practice. In this context, the possibility of providing an accurate hip fracture risk prediction tool would have a strong social and economic impact on a greying society. The so-called BBCT-hip digital twin methodology, which predicts the absolute risk of fracture for a subject upon falling starting from his QCT images, height and weight, could outperform aBMD, as demonstrated in a retrospective clinical cohort^6,28^. The first step in implementing BBCT-hip methodology consists in the QCT image segmentation to extract the patient-specific femur geometry. So far, the QCT image segmentation was carried out almost entirely manually, which is time-consuming, suffers from operator dependency, and prevents the full automatization of the BBCT-hip pipeline. Therefore, the aim of this work was to develop, implement and validate a semi-automated and unsupervised segmentation procedure. Although a plethora of supervised and automatic segmentation methodologies are currently available in the literature, these require massive, labelled dataset to be trained with, and their performance might depend on the kind of data used for training indeed. Bearing in mind that the ultimate scope would be to use the femur segmentation to predict in silico the hip fracture risk for elderly subjects, who may have a pathological anatomy of the femoral district, an unsupervised methodology was preferred. Such a methodology could also turn out useful to easily produce extensive labelled datasets, useful to train supervised fully automatic algorithms. Therefore, a semi-automatic unsupervised segmentation methodology was here presented, which, based on BJE filter and graph-cut algorithms, allows to efficiently segment the femur bone from CT scans after an operator sparsely labels a few slices highlighting bone and background. From an ethical point of view, because this pipeline is based on CT scans of living subjects, it could be directly employed on the subjects’ clinical images as long as it is run on a secured network, e.g., inside a hospital firewall. Otherwise, the CT data should be fully anonymised, which was the case of the data of the ten subjects here considered. The resulting segmentation, representing a secondary data, would not have any link to the identity of the subject it was extracted from. We compared the proposed pipeline to an almost fully manual segmentation procedure. For this purpose, 10 post-menopausal subjects were selected from the HipOp collection at Rizzoli Orthopaedic Institute. Their QCT images were segmented by the same operator adopting the two procedures. Each segmentation procedure was repeated 4 different times per subject, aiming to assess the method’s reproducibility. The median of the intra-manual metrics distributions turned out to be higher than the intra- semi-automated one for the DUR and AHD metrics, but not for the HD. HD focuses more on worst-case errors rather than considering the shapes as a whole: the two methodologies appear similar in these terms, which might be due to the same operator performing the manual segmentation and the manual refinement. From the group analysis made with the GLMM, the contribution to the model given by the random slope term turned out to be negligible for the DUR and AHD (2vs3 *p* -value > 0.05), contrary to the random intercept (3vs 4 *p*-value < 0.05). The absence of the random slope contribution might suggest that, for each patient, no significant relationship between the intra and inter-segmentation variability could be identified. This result was analogous to those obtained by the linear regression analysis performed on non-grouped data. On the other hand, the statistical significance of the random intercept in the model may indicate that the relationship between the intra and the inter-segmentation variability contained a patient dependent component. . This could be explained considering the intrinsic complexity linked to the femur anatomy and the possible presence of pathologies like osteoporosis and arthrosis, which can make the femur identification challenging. As for HD instead, both random slope and intercept appeared to be significant in the GLMM, with an intrinsic factor dependent on the specific patient. Eventually, the blind evaluation analysis performed allowed to conclude not only that the two segmentation techniques often resulted in considerably different segmentations, but also that better results were achieved by the proposed method. In spite of the geometric differences between the segmentations, however, no statistically significant differences could be identified between the two methodologies for the risk of hip fracture (ARF0), the main outcome of interest.

Analogously to the present study, several other studies presenting new segmentation techniques for the femur employed the manual segmentation as ground truth. More in detail, the results obtained herein were in good agreement with^30^, where a similar segmentation strategy was developed and comparable HD values were reported regarding the comparison between the manual and semi-automated segmentations. There, the comparison was also carried out for two FE outcomes, namely the stiffness and the strength of the implemented models, achieving, as in the here presented work, *R*^2^ values close to 1. Besler et al.^25^, who implemented an unsupervised and semi-automated segmentation pipeline similar to the presented strategy, obtained a FE-derived failure load in excellent agreement (*R*^2^ > 0.99) with that obtained starting from their ground truth as well. The comparison between the here obtained results and those shown for fully automated segmentation techniques is harder, since^11,16^ did not report any results related to FE models, although similar values were obtained by^11^ when comparing automated and manual segmentation based on the DUR and AHD geometry metrics. In general, the majority of the works where CNN-based automatic segmentation methodologies were presented achieved HD and AHD comparable to those here obtained^21,33^, although often the DUR values were lower^11,18–20,22,34^. FE-derived outcomes were reported only in some of the studies where fully automatic and supervised segmentation algorithms were presented^13,17^, although the general rationale of most of the studies was often linked to the in silico prediction of femur fracture. *R*^2^ > 0.99 were reported in^17^ when comparing the FE-derived strength between manual and automatic segmentations, while slightly lower *R*^2^ values were achieved in^13^, computed on strains. The here presented pipeline was also compared to the deep-learning technique provided in^35^, but no significant differences between the resulting segmentations emerged, as highlighted in Section 5 of the Electronic Supplementary file.

In summary, a semi-automated and unsupervised segmentation approach was here presented and provided in open access, which proved able to provide the femur segmentation with minimal intervention by the user in a few minutes. The comparison between the semi-automated and manual segmentations agreed with similar studies in the literature, and high correlation values were obtained between the hip fracture risk assessments performed by the BBCT-hip in silico methodology starting from the two segmentations. Although the method was validated on ten subjects only, the outcomes could already prove its stability and promising potential. Being semi-automated, the main shortcoming of the presented methodology is that the user intervention is required at least once, to label the CT scans. In addition, a further manual intervention might also be required at the end, to clean the obtained geometry. Although we acknowledge that the proposed methodology is not fully automated yet, the combination of the BJE filter and graph-cut algorithm would allow the future development of a fully automatic and unsupervised methodology. The here presented methodology will allow to easily segment new CT scans robustly, without any dependence on the training set as in the case of supervised techniques. It represents a step forward in fostering the adoption of in silico strategies in the clinical practice and will also allow the easier creation of large and labelled datasets to train and validate fully automated supervised methods.

### Supplementary Information

Supplementary Information.

## Data Availability

The python codes for the semiautomated segmentation linked to this manuscript are shared in Open Access at the following link: https://github.com/RiccardoBiondi/FemurSegmentation. The patients considered in the study are part of the Hip-Fracture validation collection provided in open access at https://doi.org/10.6092/unibo%2Famsacta%2F7277.
