# Supplementary material for: Development and validation of a semi-automated and unsupervised method for femur segmentation from CT
Source: Sci Rep. 2024 Mar 28;14:7403. doi: 10.1038/s41598-024-57618-6 (PMC10978861; doi:10.1038/s41598-024-57618-6)
Supplement: Supplementary file 1 — Supplementary Information. [file 41598_2024_57618_MOESM1_ESM.pdf]

A Semi-Automated and Unsupervised Method for the Segmentation of Femur CT Scans: Development and Validation on a Clinical Cohort for the Prediction of Hip Fracture Risk

Electronic Supplementary Material

Biocybernetics and Biomedical Engineering

Alessandra Aldieri, Riccardo Biondi, Antonino A. La Mattina, Julia A. Szyszko, Stefano Polizzi, Daniele Dall'Olio, Nico Curti, Gastone Castellani, Marco Viceconti

1 Graph-Cut Parameter Characterization

To better characterize the dependency of the segmentation results to the graph-cut standard deviation (σ) and weighing parameter (λ) each parameter was modified individually, while the segmentation process remained consistent with initial conditions and no manual refinement was performed. This process was repeated for every patient. The DUR was then computed by comparing each segmentation result with the reference manual segmentation. The standard deviation was adjusted within the range of 0.15-1.95 with increments of 0.15, while the weighing parameter was scaled up by a factor of 10 within the range 1-10000. For the first experiment the weighing parameter was fixed at $\lambda = 100$; for the second one the standard deviation was fixed at $\sigma = 0.25$.

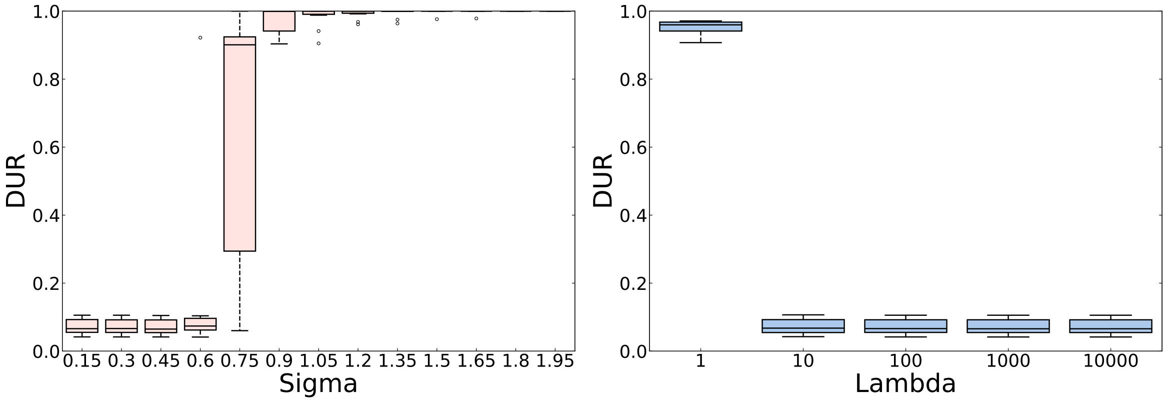

Fig. 1s : a) Boxplot representing the distribution of the DUR metric at different graph-cut standard deviation values, ranging in 0.15-1.95 across patients. b) Boxplot representing the DUR metric results at different weighing parameter values. The values range in 1-10000 and at each point the magnitude is increased by 10.

Fig. 1s displays the distribution of the DUR values at different hyperparameters values. Fig. 1s a) display the distribution obtained by changing the standard deviation(σ) parameter for the graph-cut. Notably, favorable outcomes are achieved within the range of sigma values between 0.15 and0.6. Yet, the results drastically decline for values exceeding0.75. In Fig. 1s b) display the distribution obtained by changing weighting hyperparameter for the graph-cut (λ). It can be noted that when the values exceed 1, the segmentation results do not differ significantly. However, if lambda is set to 1, the results experience a significant drop.

The semi-automate segmentation pipeline was evaluated based on two characteristic hyperparameters. The parameter σ, which represents the standard deviation for graph-cut, yielded satisfactory results within the range of [0.15, 0.60]. However, beyond this range, the segmentation quality significantly decreased due to the voxels be considered very similar in terms of boneness, making it nearly impossible to differentiate between classes. On the other hand, the weighting parameter consistently produced reliable outcomes as long as its value exceeded 10; otherwise, it led to inadequate results.

2 Supplementary figures

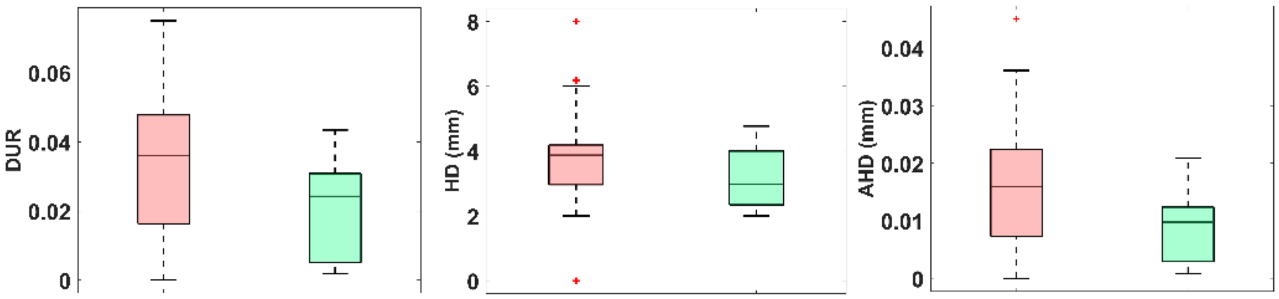

Fig. 2s : Boxplots comparing the distributions of the intra-manual (in pink) and intra-semi-automated (in light green) segmentation metrics computed: the DUR, the HD and the AHD. The median of the intra-manual metrics resulted significantly higher ($p < 0.01$).

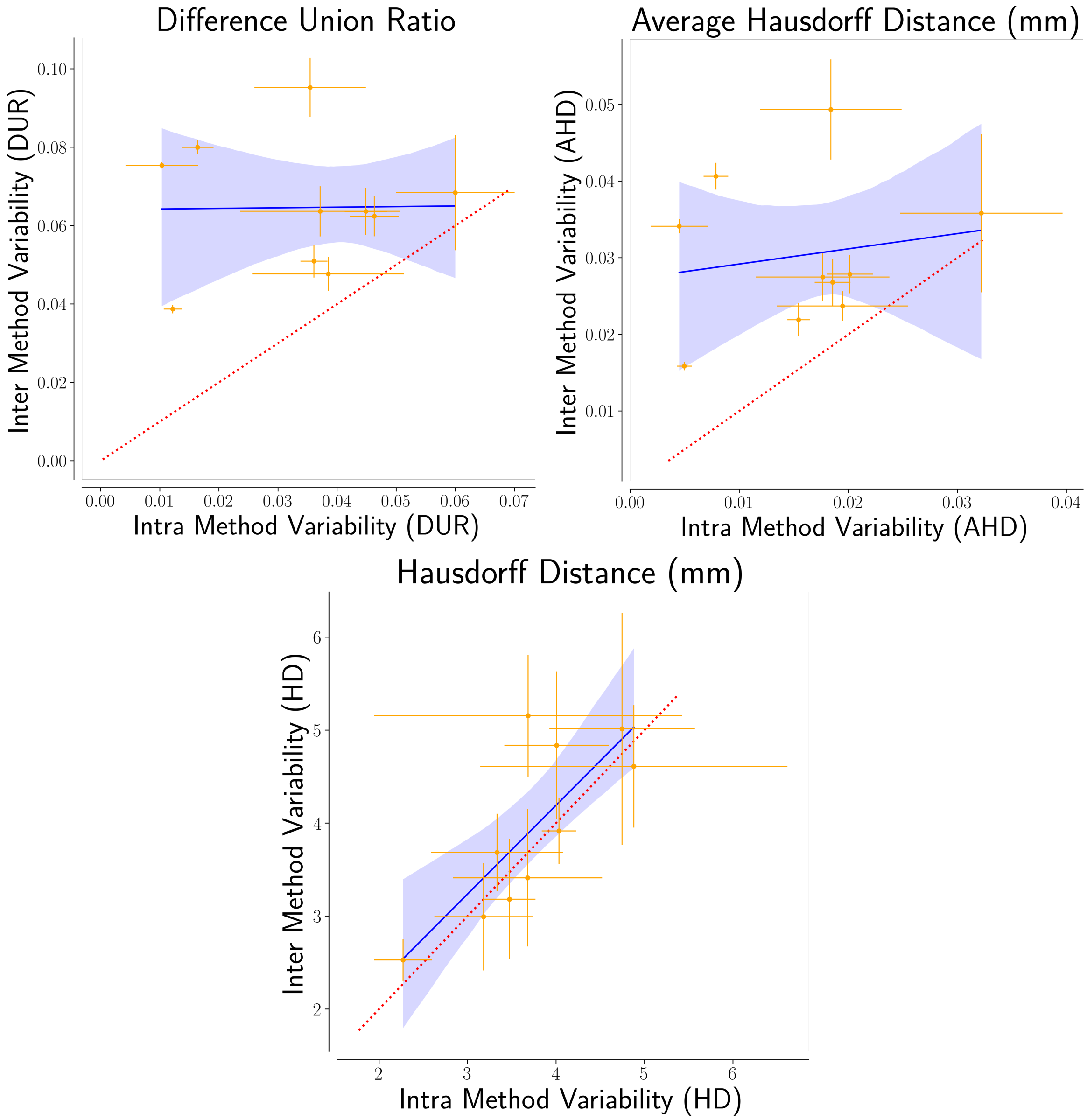

Fig. 3s : Distance metrics (DUR, AHD, HD) computed across semi-automated and manual segmentations (Inter-segmentation) plotted against the ones computed within the manual segmentations (Intra-segmentation). The error bars report the standard deviation of the estimated values. In blue the estimated regression line with the 95% confidence interval (computed by bootstrapping) is reported, in red the bisector line as reference.

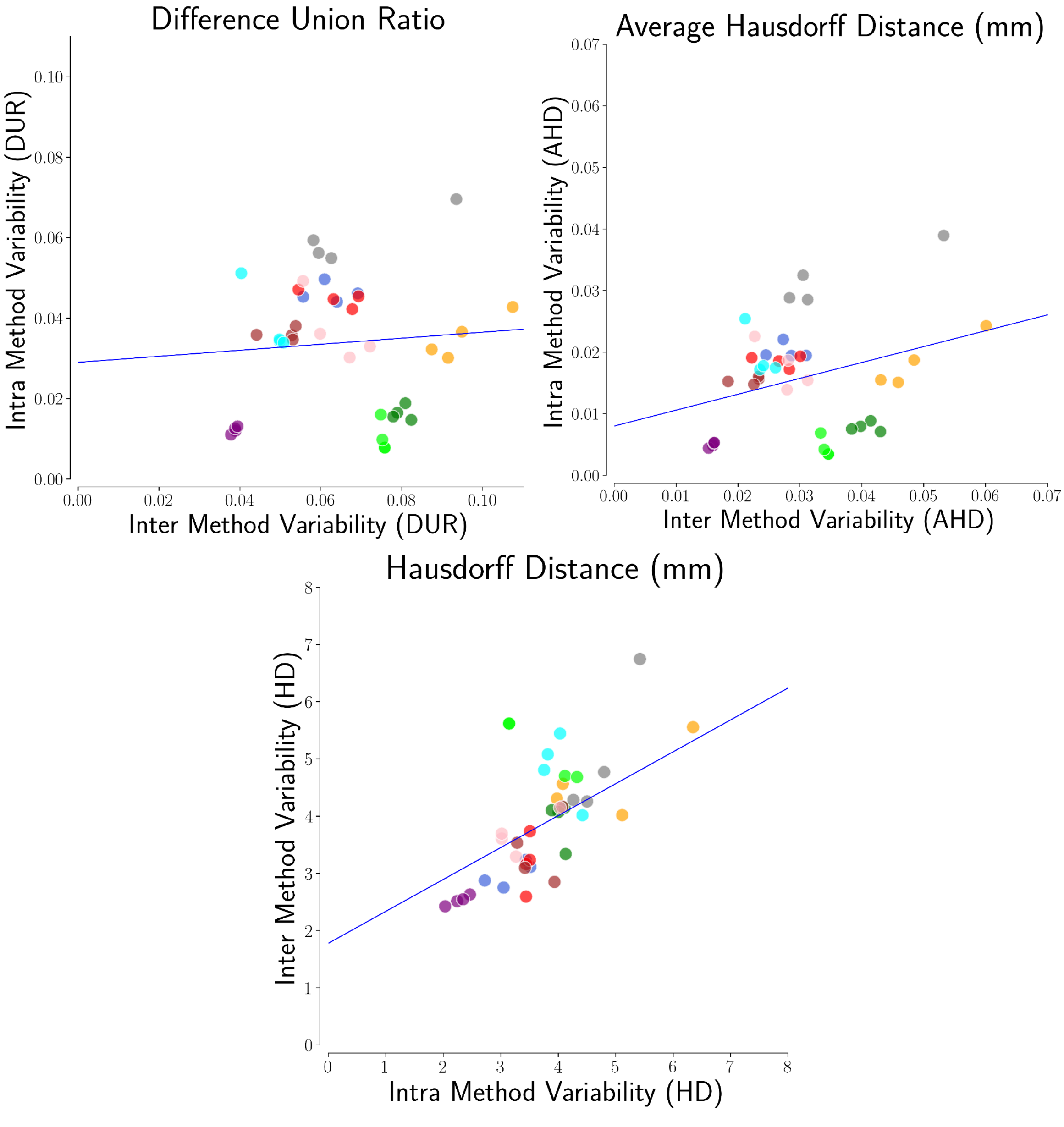

Fig. 4s : Distance metrics (DUR, AHD, HD) computed across semi-automated and manual segmentations (Inter-segmentation) plotted against the ones computed within the manual segmentations (Intra-segmentation) for all the subjects, depicted with different colours. Each ith point in the plot has been obtained as average of the distance metrics computed between the ith segmentation and all the other segmentations available.

3 Supplementary tables

In Table 1s and Table 2s are reported the values of the random effects estimated by the GLMM selected according to the likelihood ratio test: only random intercept for the Difference Union Ratio (DUR) and Average Hausdorff Distance (AHD); correlated random intercept and random slope for the Hausorff Distacne case. Namely, in Table 1s are reported the random intercept of the GLMM for the Difference Union Ratio and Average Hausdorff Distance metrics. in Table 2s are reported the random intercept and slope of the GLMM for the Hausdorff Distance metric.

Group	*DUR Random Intercept*	*AHD Random Intercept*
1	0.029	0.017
2	0.017	0.015
3	0.013	0.010
4	−0.017	−0.008
5	−0.022	−0.007
6	0.0001	−0.004
7	−0.013	−0.008
8	−0.004	−0.005
9	−0.002	−0.005
10	−0.001	−0.004

Table 1s : Estimated Random Intercept of the GLMM for DUR and AHD metric. Each group represents a single patient.

Group	*HD Random Slope*	*HD Random Intercept*
1	−0.017	0.076
2	0.005	−0.174
3	−0.103	1.346
4	−0.043	0.728
5	0.040	−0.529
6	0.032	0.194
8	0.020	−0.455
7	0.034	−0.590
9	0.032	−0.580
10	0.000	−0.016

Table 2s : Estimated random slope and intercept of the GLMM for HD metric. Each group represents a single patient.

Table 3s : The deterministic and stochastic parameters used in the fall mathematical model. For the stochastic variables the truncation points adopted to truncate their normal probability distributions are reported.

Deterministic Parameters		
$H(m)$	*Subject-specific*	
$m(kg)$	*Subject-specific*	
c	0.554	
$\Delta t(s)$	0.09	
η_{ST}	$0.0231 \times BMI - 0.33$	
k	0.5	
Fully Stochastic Parameters		
Parameter	*min*	*max*
θ_i	$0°$	$30°$
θ_f	$60°$	$120 * \circ$
$\dot{\theta}_i$	$0ms^{-1}$	$1.4ms^{-1}$
$\ddot{\theta}_i$	$0ms^{-2}$	$5.1ms^{-2}$
η_I	-2.25	0.914
η_P	0.5	0.8

4 A detailed description of the BBCT-hip methodology

BBCT-hip calculates ARF0, the absolute risk of fracture at the hip at time 0 upon falling. ARF0 is identified by calculating possible impact forces derived from a fall (through a **stochastic mathematical model**) and by assessing which of those, exceeding the load to failure (determined through a **patient-specific FE model**), lead to a fracture event.

4.1 Impact force calculation

BBCT-hip methodology computes the impact load which results from a sideways fall from standing. Reasonably, it is not possible to determine the precise impact load one person will experience during a fall in a deterministic way, since one person may fall in very different ways. This is the reason why the impact force definition is based on a **stochastic mathematical model**. This model is a physics-based mathematical model which is also defined as stochastic because some of the variables it includes cannot be deterministically defined and are therefore described by probability distributions which are sampled to compute possible individual impact forces. The fall is idealized as an inverted pendulum: a rotation of the whole body on any plane containing the vertical axis (fall plane). The rotation occurs around a spherical joint (hinge) fixed to the floor and located near the foot on the side of impact. The model considers the body mass (m) to be concentrated at the moving end of the inverted pendulum, the static end of which is located at the hinge. The pendulum length (h) equals the body Centre-Of-Mass (COM) elevation from the ground in the upright position. It is taken to be a fixed proportion ($c = 0.554$) of the subject's standing height (H) [2]. BBCT-hip calculates the impact force caused by a sideways fall as follows:

$$F = (m\frac{\sqrt{2(1-\eta_P e)}}{\Delta t})(1-\eta_I)(1-\eta_{ST}) \tag{1}$$

with

$$e = c^2H^2\ddot{\theta}_i(\theta_f - \theta_i) + \frac{1}{2}c^2H^2\dot{\theta}_i^{\,2} + gcH(cos(\theta_i) - cos(\theta_f))) \tag{2}$$

θ_i and θ_f represent the initial and final inclinations of the body with respect to the vertical, respectively, $\dot{\theta}_i$ and $\ddot{\theta}_i$ initial angular velocity and angular acceleration. η_P is the postural reflex attenuation coefficient, η_I accounts for damping due to flooring elements, hip protector devices (if present), and active soft tissues (muscles) that may contract at the instant of impact. η_{ST} instead, accounts for damping due to all passive soft tissues interposed between the point of impact on the skin and the lateral aspect of the greater trochanter. Δt represents the total impact duration. All the variables included in the aforementioned mathematical formulations, which allow the extraction of the impact force, are listed in the following Table 3s. For the impact forces estimation, the only subject-specific variables, therefore, are weight and height.

BBCT-hip computes a total of $1,000,000$ falls of a body of the height and weight equal to that of the patient. To do so, inverse latin hypercube is employed each time to sample over the 6 stochastic input variables: patient

initial and final position (θ_f, θ_i), initial speed and acceleration ($\dot{\theta}_i$, $\ddot{\theta}_i$), damping coefficients (η_P, η_I) reported in Table Vs. For each simulated fall, the resulting impact force (F_I) is hence calculated.

4.2 Load to failure prediction

BBCT-hip uses a patient-specific finite element model of the femur informed by the patient's QCT data to predict the biomechanical deformation and from deformation the force required to fracture the femur (load to failure). The patient-specific finite element model is built according to the following procedure.

4.2.1 Geometry extraction from CT images

Patient CT scans are segmented in order to extract the patient-specific femur geometry from the images. Anatomical landmarks (placed at the centre of the femur head, distal femur epicondyles most medial and lateral points, distal femur medial and lateral epicondyles most posterior points) are identified for the definition of an anatomical reference system used in the application of the boundary conditions replicating a fall (Fig. 5s).

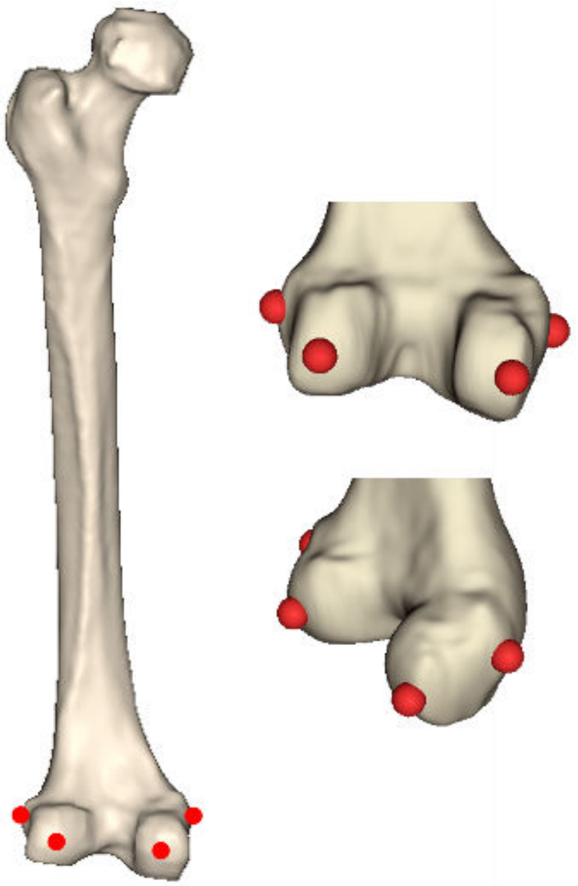

Fig. 5s : The landmark positioned in the epicondylar region.

More in detail, the femur head centre is identified by fitting the femur head surface with a sphere, while distal epicondyles points are chosen by the operator. The local reference system is created as follows: the knee rotation centre is calculated as the mean location between most medial and most lateral epicondyle points; the reference system origin is placed in the femur head centre, and the X axis is directed towards the knee rotation centre; the Y axis is directed towards the greater trochanter, with direction identified by posterior condyles coordinates (automatic orthogonalization performed by ANSYS Mechanical APDL); the Z axis is consequently calculated.

4.2.2 Mesh creation and material properties assignment

The femur geometry is automatically meshed with a linear 3D tetrahedral mesh (Octree method, max element size 2 mm following sensitivity analysis); mid-side nodes are added, and the mesh is converted into 10-node quadratic tetrahedrons. Afterwards, HU-based mechanical properties are assigned using Bonemat software [5] to map local bone material properties elementwise, i.e. to assign HU-based material properties to the mesh elements according to [3].

Where E_l is the Young's modulus (MPa) and ρ_{app} the apparent density (computed from the ash density ($\rho_{app} = \frac{\rho_{ash}}{0.6}$ whith $\rho_{ash} = 0.877\rho_{QCT} + 0.079$) [4]).

For each finite element model, the mesh elements are grouped according to their HU as averaged from the voxels they contain, and for each group, the same elastic modulus is assigned. The HU range on which grouping is performed is 50 HU.

4.2.3 Loading and Boundary conditions

The load is applied at the femur head centre, and a rigid frictionless contact plane perpendicular to the load direction is created at the greater trochanter to replicate the ground, at the most lateral node with respect to the load direction; a revolute joint is placed at the knee rotation centre, with the rotation axis along the antero-posterior direction, perpendicular to the load axis; distal diaphysis (a 4 mm thick bands of nodes at 75% of the biomechanical length, from femur head towards knee is selected) is constrained to rotate around knee centre (Fig. 6s).

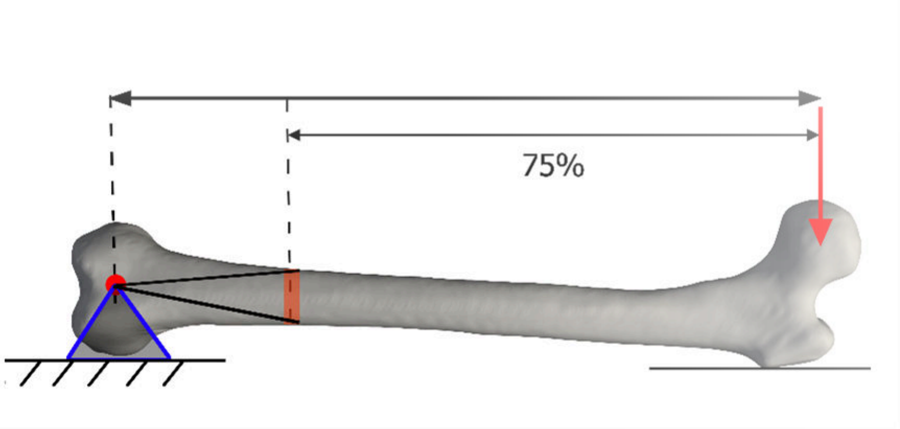

Fig. 6s : Schematic of the applied boundary conditions and loading. The femur orientation is exemplificative. The red arrow represents the applied load, and the black one the biomechanical length.

The proximal femur surface nodes (25% of the biomechanical length in the longitudinal direction) are selected as the region of interest for the load to failure determination (Fig. 7s).

The biomechanical deformations of bone vary linearly with the biomechanical stresses up to deformation of 0.8-1.0%. During hip fractures, at that level of deformation, a crack propagates macroscopically to fracture in a few milliseconds. In such cases, the prediction of the strength can be done with good accuracy by simply considering the elastic properties of the bone tissue.

Hence, BBCT-hip calculates the load to failure, i.e. the load supposed to cause a fracture, based on the biomechanical deformation in the ROI. The load to failure is identified as the load applied to the femur head which makes principal deformation exceed specific thresholds (0.73% and -1.04% for the first and third principal deformation respectively) [1]. The simulations are run in ANSYS Mechanical APDL with an automated macro script (Ansys 2019 R3 version). The model is solved with a large deflection option on and the automatic time stepping for the Newton-Raphson scheme available in Ansys for Augmented-Lagrangian contact problems (ANSYS, Inc. Help Guide). All simulations were solved using the preconditioned conjugate gradient-iterative solver (PCG) with a tolerance value of 1×10^{-8}. Because the femur can impact the ground with different orientations, multiple finite element simulations need to be performed on the same model varying the loading condition and predicting the load to failure for each. This implies that the impact load direction is varied from $0°$ to $30°$ in the frontal plane (adduction) and $-30°$ to $+30°$ in the transverse plane (internal-external rotation) in an anatomical reference system (Fig. 8s).

This range covers falls on both anterolateral and posterolateral directions. The lateral angles were not considered as this fall direction was not plausible because within this orientation the knee will the ground but not the hip.

The prediction of the load to failure for any possible direction comprised in these ranges would require running an extremely high number of times the full-order finite element model, demanding an excessive computational cost. Therefore, a reduced-order model (response surface) is used to predict the load to failure for each possible impact direction running the full-order finite element model only 28 times. A total of 28 different finite simulations are run where the impact direction is varied sampling the full angle range in steps of $10°$. Therefore, a response surface as the one shown in Fig. 9s is built by linearly interpolating the points defined by the 28 different loads to failure obtained from the full-order finite element models.

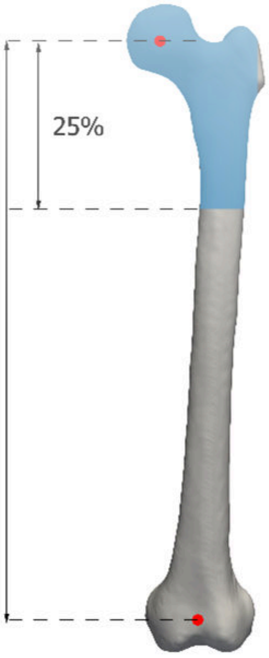

Fig. 7s : In blue the Region Of Interest (ROI) where the strain-based fracture criteria are evaluated is displayed. The most lateral part of the greater trochanter is excluded due to boundary conditions effects. The black arrow indicates the biomechanical length.

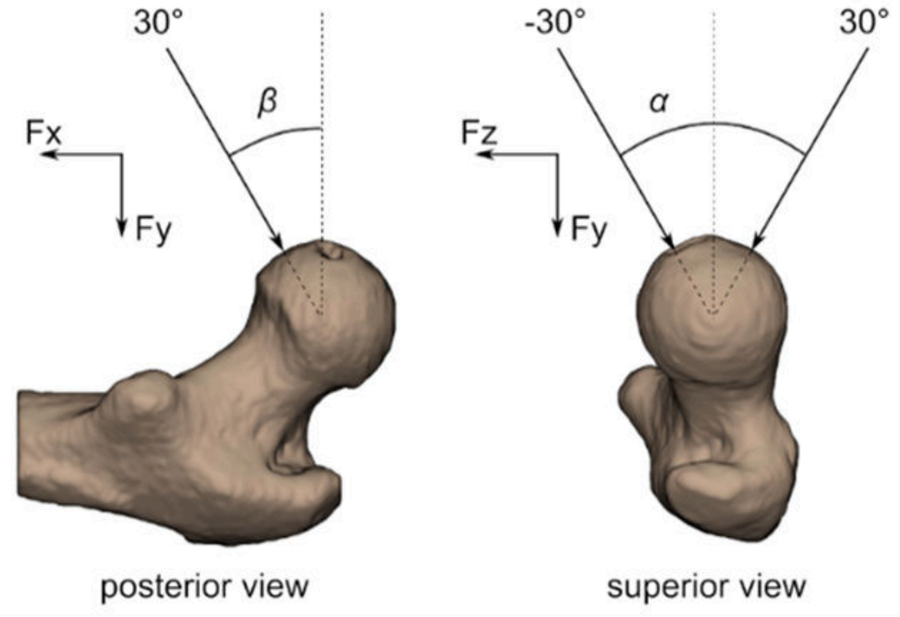

Fig. 8s : The reference system with the angles α and β highlighted.

4.2.4 ARF0 calculation

BBCT-hip computes ARF0 by determining and comparing the 1,000,000 predicted fall impact forces F with the load to failure response surface (e.g. Fig. 12s), those which would lead to a femur fracture. For each of the one million impact forces, we compare such force to the force required to fracture the femur in the given pose of impact (load to failure). P is merely the number of simulated falls for which the model predicts fracture, divided by the total number of simulated falls. Mathematically, $n = 1, \cdots, N$ is the fall number, i.e. one of N total number of simulated falls. $I(n)$ is the force that impact transmits to the femur for fall n. F is the force required to fracture the femur, which varies as a function of the femoral orientations. We assume a fall cause fracture if $I(n) > F$. NF is the number of simulated falls that cause a fracture. $ARF0 = \frac{NF}{N}$ is the absolute

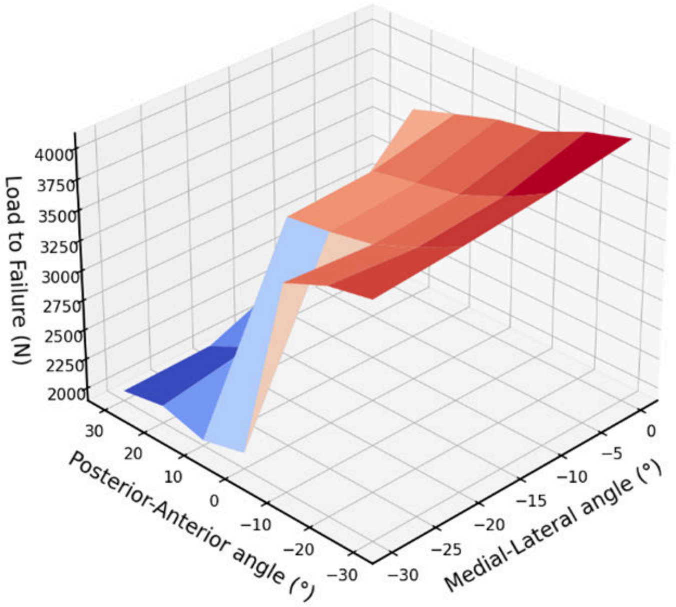

Fig. 9s : The response surface built on the failure to load values obtained from the 28 impact directions simulated with the full order finite element model.

risk of fracture.

5 Comparison with Deep-Learning fully automated methods

The semi-automated method developed was compared with a deep-learning-based segmentation method by Wasserthal et al. [6]. The method, known as "Total Segmentator" is not specific to femur segmentation but can identify multiple anatomical regions. The comparison was conducted using manual segmentation results as a reference and utilizing the difference union ratio (DUR) as a similarity metric. Additionally, we analyzed the distribution total volume (in term of number fo voxels) identified by each method. In order to assess if the obtained distributions are significantly different between each other, wilcoxon test was performed.

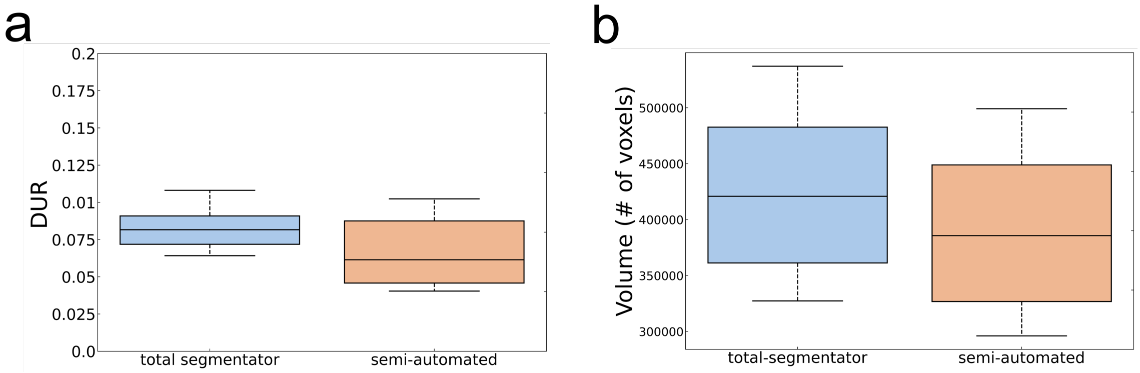

Fig. 10s : a) Distribution of the Difference Union Ratio computed against the manual segmentation for both the semi-automated and total-segmentator segmentation approaches. b) segmented femur volume (number of voxels) distribution for both the semi-automated and total-segmentator segmentation approaches.

**Method**	**Metric**	**Median**	**IQR**
semi-automated	DUR	0.061	0.088
semi-automated	Volume	385702	122168
total-segmentator	DUR	0.082	0.019
total-segmentator	Volume	420873	121462

Table 4s : Estimated Random Intercept of the GLMM for DUR and AHD metric. Each group represents a single patient.

In tab. 4s are reported median and IQR for each distribution in fig. 10s. The DUR metric distribution is shown in fig. 10s a. It can be observed that the semi-automated method achieves a segmentation closer to the manual one compared to the total segmentator. Conversely, the total segmentator appears to yield more consistent results, with a lower interquartile range (IQR) of the metric distribution. The Wilcoxon test conducted between the two distributions does not yield significance ($p\_value = 0.19$), indicating that both methods show equal similarity to the manual segmentation.

Fig. 10s b displays the distribution of volumes segmented using both methods in terms of voxels. It can be observed that the volume segmented with the semi-automated method is lower ($median = 385702$) compared to the one obtained by the total segmentator ($median = 420873$). Additionally, the interquartile ranges are very similar. The Wilcoxon test yielded a p-value of 0.001, confirming that the volume obtained by the semi-automated method is significantly lower than the one obtained by the total segmentator.

The semi-automated segmentation method outperforms the manual method in terms of contour fidelity. This suggests that the segmentation obtained with the total segmentator is less accurate in delineating edges compared to the semi-automatic segmentation, leading to an overestimation of femur volume.

References

[1] Harun H Bayraktar, Elise F Morgan, Glen L Niebur, Grayson E Morris, Eric K Wong, and Tony M Keaveny. Comparison of the elastic and yield properties of human femoral trabecular and cortical bone tissue. *J. Biomech.*, 37(1):27–35, January 2004.

[2] Marguerite I Croskey, Percy M Dawson, Alma C Luessen, Irma E Marohn, and Hazel E Wright. The height of the center of gravity in man. *Am. J. Physiol.*, 61(1):171–185, June 1922.

[3] Elise F Morgan, Harun H Bayraktar, and Tony M Keaveny. Trabecular bone modulus-density relationships depend on anatomic site. *J. Biomech.*, 36(7):897–904, July 2003.

[4] Enrico Schileo, Enrico Dall'ara, Fulvia Taddei, Andrea Malandrino, Tom Schotkamp, Massimiliano Baleani, and Marco Viceconti. An accurate estimation of bone density improves the accuracy of subject-specific finite element models. *J. Biomech.*, 41(11):2483–2491, August 2008.

[5] Fulvia Taddei, Enrico Schileo, Benedikt Helgason, Luca Cristofolini, and Marco Viceconti. The material mapping strategy influences the accuracy of CT-based finite element models of bones: an evaluation against experimental measurements. *Med. Eng. Phys.*, 29(9):973–979, November 2007.

[6] Jakob Wasserthal, Hanns-Christian Breit, Manfred T. Meyer, Maurice Pradella, Daniel Hinck, Alexander W. Sauter, Tobias Heye, Daniel T. Boll, Joshy Cyriac, Shan Yang, Michael Bach, and Martin Segeroth. TotalSegmentator: Robust Segmentation of 104 Anatomic Structures in CT Images. *Radiology: Artificial Intelligence*, 5(5):e230024, September 2023.
